# Genetic analysis of the *PCSK9* locus in psychological, psychiatric, metabolic and cardiovascular traits in UK Biobank

**DOI:** 10.1038/s41431-022-01107-9

**Published:** 2022-05-02

**Authors:** Rachel Hay, Breda Cullen, Nicholas Graham, Donald M. Lyall, Alisha Aman, Jill P. Pell, Joey Ward, Daniel J. Smith, Rona J. Strawbridge

**Affiliations:** 1 Institute of Health and Wellbeing, University of Glasgow, Glasgow, UK; 2 Institute of Cardiovascular and Medical Sciences, University of Glasgow, Glasgow, UK; 3 Centre for Clinical Brain Sciences, University of Edinburgh, Edinburgh, UK; 4 Health Data Research UK, Glasgow, UK; 5 Cardiovascular Medicine Unit, Department of Medicine Solna, Karolinska Institute, Stockholm, Sweden

**Keywords:** Risk factors, Genetics, Molecular biology, Cardiovascular diseases, Psychiatric disorders

## Abstract

The association between severe mental illness (SMI) and cardiovascular and metabolic disease (CMD) is poorly understood. *PCSK9* is expressed in systems critical to both SMI and CMD and influences lipid homeostasis and brain function. We systematically investigated relationships between genetic variation within the *PCSK9* locus and risk for both CMD and SMI. UK Biobank recruited ~500,000 volunteers and assessed a wide range of SMI and CMD phenotypes. We used genetic data from white British ancestry individuals of UK Biobank. Genetic association analyses were conducted in PLINK, with statistical significance defined by the number of independent SNPs. Conditional analyses and linkage disequilibrium assessed the independence of SNPs and the presence of multiple signals. Two genetic risk scores of lipid-lowering alleles were calculated and used as proxies for putative lipid-lowering effects of PCSK9. *PCSK9* variants were associated with central adiposity, venous thrombosis embolism, systolic blood pressure, mood instability, and neuroticism (all *p* < 1.16 × 10^−4^). No secondary signals were identified. Conditional analyses and high linkage disequilibrium (*r*^*2*^ = 0.98) indicated that mood instability and central obesity may share a genetic signal. Genetic risk scores suggested that the lipid-lowering effects of *PCSK9* may be causal for greater mood instability and higher neuroticism. This is the first study to implicate the *PCSK9* locus in mood-disorder symptoms and related traits, as well as the shared pathology of SMI and CMD*. PCSK9* effects on mood may occur via lipid-lowering mechanisms. Further work is needed to understand whether repurposing PCSK9-targeting therapies might improve SMI symptoms and prevent CMD.

## Introduction

It has been long recognised that severe mental illness (SMI) is accompanied by an increased burden of cardiovascular and metabolic diseases (CMD) [1, 2]. The direct cause of this comorbidity remains ambiguous; however, it is likely influenced by factors such as genetics, socioeconomic status, poor diet, sedentary lifestyle, substance abuse and side effects of psychiatric medication [3]. Additionally, genetic studies have begun to provide evidence of shared mechanisms underlying mental and physical ill-health [4–7].

For many years CMD research has focused on *PCSK9* and its encoded protein (also PCSK9), because of its key role in lipid homeostasis. Specifically, PCSK9 binds to the low-density lipoprotein (LDL) receptors (LDLR) and targets them for degradation [8]. Lipid homeostasis is important for energy storage and release and is therefore central to metabolic processes. The balance of LDL in circulation vs in tissues is important, as high circulating levels of lipids can lead to inappropriate storage of lipids, which contribute to insulin resistance and systemic inflammation observed in CMD [9]. PCSK9 has direct effects on pancreatic cells and adipose tissue, which are critical organs for maintenance of insulin sensitivity, with lipid-lowering and non-lipid-lowering mechanisms (respectively) implicated [10]. In particular, deposition of lipids in blood vessel walls is a key feature and risk marker of cardiovascular diseases including myocardial infarction and ischemic stroke. High levels of circulating LDL have a causal relationship with atherosclerotic plaques [11] and the importance of PCSK9 in these processes is highlighted by the development of PCSK9 inhibitors as a treatment of atherosclerotic disease. Clinical trials have demonstrated the benefits (and importantly no side-effects) of lipid-lowering via increased LDLR activity (reviewed in [12]). In addition to lipid-lowering, PCSK9 has direct effects on platelet activation and thrombosis risk [13], which have been prevented or reduced with PCSK9 inhibitors, experimentally [12] and clinically [14].

PCSK9’s association with SMI remains less established, however an association seems plausible due to its roles in brain functions [15, 16]^,^, which include brain cholesterol trafficking and lipoprotein trafficking [17] and insulin resistance, which is implicated in SMI [18]. Indeed, there is evidence that depressive symptoms alone can raise PCSK9 levels, with a subsequent effect on insulin resistance [19]. Under some circumstances, lipids can cross the blood brain barrier, and as well as influencing lipid regulation, PCSK9 is expressed in areas with high proliferative ability. It has a positive correlation with increased post-mitotic neurones and decreased neuroepithelial cells. Additionally, it has been shown to promote or prevent apoptosis in many neuronal pathways. This emphasises the influence of PCSK9 on neurogenesis and neuronal apoptosis. Finally, inhibition of *PCSK9* is associated with reduced neuroinflammation. PCSK9 has been investigated in neurodegenerative disorders such as Alzheimer’s disease, where altered levels of the protein levels in cerebrospinal fluid (CSF) [17] and brain samples [20] have been associated with disease. PCSK9 levels have also been reported to be upregulated in alcohol use disorders or ischemic stroke [15, 20]. Clinical studies demonstrate that, despite some initial concerns, PCSK9 inhibitors do not have adverse effects on cognition [21], although rate of adverse psychiatric reactions due to lipid-lowering (by either PCSK9 inhibition or non-PCSK9 methods such as statins) requires further examination [22].

A number of studies exploring the genetic variants in the PCSK9 locus has been conducted: One explored a single missense variant, rs11591147 (R46L), as an indicator of potential long-term effects of PCSK9 inhibition on a large number of phenotypes [23], however there is evidence that the locus contains more than one independent signal [24], thus a more extensive assessment of the locus is warranted. Another genetic study investigated a number of loss-of-function variants on cognition and Alzheimer’s disease, however this study was of limited size (total *N* = 878) [25]. A further study (*N* = 2487) identified sex-specific effects of two variants in the *PCSK9* locus on CSF PCSK9 levels [20].

**Fig. 1:**
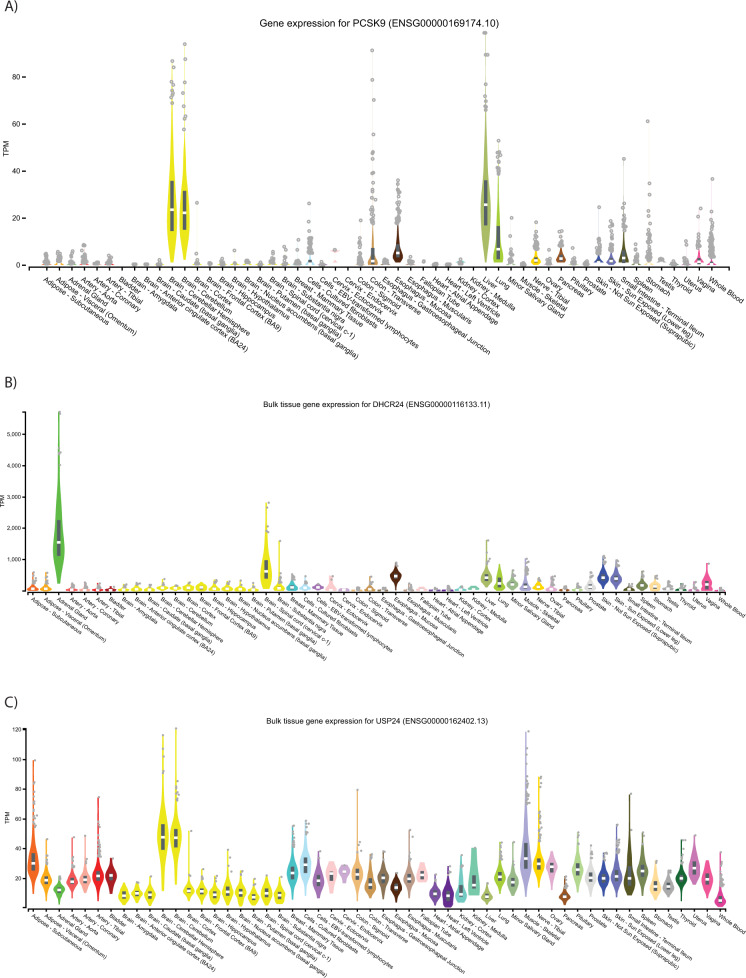
Tissue expression patterns of genes of interest. Gene expression of **A** *PCSK9*, **B** *DHCR24*, and **C** *USP24*, by tissue (GTEx analysis release V8). Expression values are shown in transcription per million (TPM) calculated from a gene model with isoforms collapsed to a single gene. Box plots are shown as median and 25th and 75th percentiles; points are displayed as outliers if they are above or below 1.5 times the interquartile range.

Given that the expression of PCSK9 is observed in the brain as well as the liver (Fig. 1), and the known functions of PCSK9, we aimed to systematically investigate whether genetic variation in the *PCSK9* locus contributes to the shared genetic predisposition and underlying pathological mechanism of both CMD and SMI. Furthermore, we describe the genetic architecture in the *PCSK9* locus and identify mechanisms by which genetic variants in this region may influence biological traits. Finally, we evaluate whether the effects of *PCSK9* genetic variation were likely to act through LDLR-lowering pathways. If *PCSK9* variation contributes to SMI by the same mechanisms as its effect on CMD, there is the potential to repurpose CMD therapies for the improvement of SMI symptoms and CMD prevention in these high CMD-risk individuals.

## Methods

### UK biobank

The UK Biobank (UKB) is a large dataset of 502,613 participants, which has generic ethical approval granted by the NHS National Research Ethics Service (approval letter dated dated 29th June 2021, Ref 21/NW/0157). This work was carried out using UKB application #6553 (PI Rona Strawbridge) and #17689 (PI Donald Lyall). Data is provided by participants and collected by the National Health Service as part of their care and support. A more thorough detailed description of the UKB data set can be found elsewhere in open-access reports [26, 27].

UKB volunteers were recruited through 22 assessment centres during 2006–2011. Individuals were largely aged between 40–69 at baseline. Trained clinical staff carried out physical examinations and touchscreen questionnaires to gather baseline measurements along with individual and family medical history. Blood samples allowed biomarker analysis and DNA extraction for genome-wide genotyping. The genotyping, quality control and imputation used standard protocols and took place centrally.

Average systolic and diastolic blood pressure reading was calculated, each from two measurements, and adjusted for the use of antihypertensive medications as per Ehret et al. [28], prior to analysis (SBPadj or DBPadj). Height and weight were recorded and BMI (kg/m^2^, reflective of total obesity) calculated. Waist and hip measurements allowed for calculation of waist:hip ratio (reflective of central obesity) which was adjusted for BMI (WHRadjBMI, reflecting central obesity accounting for total obesity) prior to analysis as per Shungin et al. [29]. Type 2 diabetes status was defined as per Eastwood et al. [30] (from self-reported medical history and medication data). Reported medical histories included venous thromboembolism (VTE), stroke, and ischaemic heart disease (ISH). Self-reported smoking was categorised as current smokers vs. non or former smokers.

Psychiatric traits were analysed by different baseline questionnaires. Firstly, the 12 items of the Eysenck Personality Questionnaire-Revised Short form were used to assess neuroticism. Lack of enthusiasm in the last two weeks was recorded to assess individual’s anhedonia (data field 2060). Individuals responding “not at all” were considered controls, whilst those responding “several days”, “more than half of the days” or “nearly every day” were considered cases. Risk-taking was assessed by a self-reported question asking, “Would you describe yourself as someone who takes risks?” (data field 2040) and mood instability by the question “Does your mood often go up and down?” (data field 1920). Individuals responding “yes” were considered cases, and those responding “no” were considered as controls. Any individual responding “prefer not to answer”, or “don’t know” was excluded from the analysis. A follow-up online mental health questionnaire (collected 2016–2017) was used to clarify the lifetime history of SMI [31, 32] and related traits. This included generalised anxiety disorder (LifeGAD), major depressive disorder (LifeMDD), bipolar disorder (LifeBD), or addiction [31, 32].

### SNP selection

The *PCSK9* locus is defined as the *PCSK9* gene ±250 kb (UCSC GRCh37/hg19, Chromosome 1:55,505,221-55,530,525 ± flanking regions). Initially all single nucleotide polymorphisms (SNPs) within the locus were identified. Discovery analyses included only unrelated individuals of white British ancestry (*N* = 402,820, SNP-N = 2037). Secondary analyses were conducted in unrelated individuals of white European ancestry (*N* = 50,510, SNP-N = 2017), south Asian ancestry (*N* = 7726, SNP-N = 2167), African-Caribbean ancestry (*N* = 7644, SNP-N = 3386) and mixed ancestry (*N* = 10,447, SNP-N = 2532). Genetic variants with minor allele frequencies of >1% by ancestry group were analysed.

### Statistical analyses

Minitab 19.2 was to calculate descriptive statistics. For genetic analyses, an additive model was assumed and Plink 1.07 [33] was used with logistic or linear regression. Continuous variables were assessed for normality prior to analysis. All analyses were adjusted for age, sex, eight genetic principal components and genotyping chip, except WHRadjBMI where these covariates are included in its construction. Treatment with lipid-lowering and anti-hypertensive medications were included as covariates for analysis of ISH and stroke. The number of independent loci was determined using independent pairwise filtering using default parameters in PLINK 1.07, and Bonferroni correction for multiple testing (using number of SNPs) was applied. The threshold for significance in discovery analyses was *p* value <1.16 × 10^−4^ and in secondary analyses, *p* value <1.28 × 10^−4^ for South Asian, <4.69 × 10^−5^ for African-Caribbean, <1.20 × 10^−5^ for white European and <8.85 × 10^−5^ for mixed ancestry individuals. LocusZoom [34] was used to generate regional plots.

### Conditional analysis: number of signals

Conditional analyses were used to identify multiple independent signals. Analyses of traits with significant associations were repeated including the lead SNP as a covariate (for example, analysis of SNPs vs BMI were adjusted for age, sex, eight genetic principal components, genotyping chip and the lead BMI SNP). An additional independent signal was defined as at least one SNP meeting the threshold for significance in the conditional analysis (as well as the original analysis). Where an additional signal was identified, a further round of conditional analysis was conducted, including the lead SNPs from both signals as covariates. This process was repeated until no further significant signals remained.

### Conditional analysis: independence of signals

Conditional analyses were also used to determine whether the signals for each significant trait were independent. Here the analyses of traits with significant associations were repeated, with the addition of the lead SNP for a different trait as a covariate (for example, analysis of SNPs vs BMI were adjusted for age, sex, eight genetic principal components, genotyping chip and the lead VTE SNP). Lead SNPs were considered independent if the effect size (Beta or OR) changed by <0.05 (arbitrary value, but consistent with the maximum magnitude of change likely with fluctuations in sample size due to inclusion of a SNP as a covariate). No distance measure was used to assess independence. Linkage disequilibrium (LD) between lead SNPs was assessed using a randomly selected 10,000 unrelated white British ancestry individuals from UKB. Haploview [35] allowed visualisation of the LD between the lead SNPs, to complemented the conditional analyses.

### Effect of Lead SNPs on PCSK9 levels

The impact of lead SNPs on circulating *PCSK9* levels was investigated using publicly available genome-wide meta-analysis summary statistics [24].

### PCSK9 polygenic scores

Three studies have highlighted genetic variants with functional effects that can be used to proxy lifelong reduction in LDL levels {Schmidt, 2019 #275;Ference, 2016 #274;Lyall, 2021 #273}. Schmidt et al. used four SNPs in a polygenic score (PGS) {Schmidt, 2019 #275}, whilst Ference et al. selected seven SNPs {Ference, 2016 #274}. Lyall et al. used six of the seven Ference SNPs and noted that individual SNPs had little impact when removed {Lyall, 2021 #273}. We constructed two PGS, using four variants (PGS4 {Schmidt, 2019 #275}) or seven variants (PGS7 {Ference, 2016 #274}), by summing the lipid-lowering alleles for each individual. Only individuals with complete genotyping for the four of seven SNPs were included.

### Follow-up analyses

The GTEx portal (https://www.gtexportal.org/home/ [36], accessed 2021-08-26) was used to explore *PCSK9* gene expression and genotype-specific gene expression patterns. LDExpress was used to explore genotype-specific gene expression patterns of proxies of lead SNPs (https://ldlink.nci.nih.gov/?tab=ldexpress [37], 2021-08-26). The GWAS Catalog, (https://www.ebi.ac.uk/gwas/, 2021-08-17), was used to identify previous associations in the *PCSK9* locus. Ensembl VEP (https://www.ensembl.org/info/docs/tools/vep/index.html, [38]) was used to explore the predicted functional effects of a SNP, with SIFT and PolyPhen being used to assess tolerance. The Human Protein Atlas (https://www.proteinatlas.org/, [39]) was used to investigate PCSK9 protein expression.

## Results

Table 1 provides the cohort characteristics. The average age of participants was 56.9 years, with 20.6% being treated with lipid-lowering medication and rates of ISH and stroke were low (6.1% and 2.1% respectively).

**Table 1:** Characteristics of the UK Biobank cohort.

	White British	White European	South Asian	African-Caribbean	Mixed ancestry
*N* (% male)	402,820 (46.0)	50,510 (43.8)	7726 (53.9)	7644 (43.0)	10,447 (42.8)
Age (years)	56.9 (8.0)	55.6 (8.1)	53.4 (8.5)	51.9 (8.1)	52.4 (8.1)
BMI (KG/m^2^)	27.4 (4.8)	27.2 (4.9)	27.3 (4.5)	29.5 (5.4)	27.0(4.9)
WHR	0.87 (0.09)	0.9 (0.09)	0.9 (0.09)	0.9 (0.1)	0.9(0.1)
SBP (mmHg)	138 (19)	135.5 (18.5)	134.9 (18.6)	137.8 (18.7)	133.4 (18.6)
DBP (mmHg)	82 (10)	81.5 (10.2)	82.5 (10.2)	84.5 (10.7)	81.9 (10.5)
SBP^a^ (mmHg)	141 (21)	138.2 (20.5)	139.1 (21.4)	142.5 (21.7)	136.4(21.0)
DBP^a^ (mmHg)	84 (11)	83.3 (11.3)	85.2 (11.5)	87.7 (12.4)	83.9 (11.9)
Current smoking	40,618 (10.1)	6597 (13.1)	687 (8.9)	952 (12.5)	1437 (13.8)
Hypertension	205,846 (51.1)	22,787 (45.1)	3832 (49.6)	4321 (56.5)	4523(43.3)
Anti-hypertensive medication	83,951 (21.0)	9235(18.3)	2082 (26.9)	2340 (30.6)	2048(19.6)
Lipid-lowering medication	70,184 (20.6)	8042 (15.9)	2049 (26.5)	1212 (15.9)	1807(17.3)
PGS4	1.39 (0.97)				
PGS7	3.88 (1.52)				
ISH	18,383 (6.1)	2135 (4.2)	575 (7.4)	264 (3.5)	414 (4.0)
Stroke	6150 (2.1)	708 (1.4)	128 (1.7)	118 (1.5)	94 (0.9)
VTE	10,444 (3.7)	1239 (2.5)	131 (1.7)	185 (2.4)	189 (1.8)
T2D	17,420 (4.3)	2121 (4.2)	1288 (16.7)	812 (10.6)	865 (8.3)
Anhedonia	78,879 (19.6)	11,255 (22.3)	2813 (36.4)	2389 (31.3)	3336 (31.9)
Mood instability	177,994 (45.2)	22,089 (43.7)	2778 (48.9)	3724 (48.7)	4754 (45.5)
neuroticism score	1.65 (5.90)	1.39 (6.11)	−0.73 (7.06)	−0.25 (6.44)	−0.28 (6.79)
Risk-taking	98,435 (25.3)	16,240 (32.2)	2688 (34.8)	2986 (39.1)	3705 (35.5)
Mental Health questionnaire	130,370 (32.4)	16,751 (33.2)	1043 (13.5)	1104 (14.4)	2277 (21.8)
Addiction^b^	7471 (5.7)	1280 (2.5)	46 (0.6)	64 (0.8)	167 (1.6)
GAD^b^	9096 (10.1)	1282 (2.5)	74 (1.0)	54 (0.7)	167 (1.6)
BD^b^	1873 (1.5)	324 (0.6)	24(0.3)	24 (0.3)	51 (0.5)
MDD^b^	30,875 (28.1)	4186 (8.3)	196 (2.5)	203 (2.7)	539 (5.2)

Continuous variables are presented as mean (se), binary variables are presented as *N* (%).

*WHR* Waist: hip ratio, *PGS4* Polygenic score including four SNPs as per Schmidt et al, *PGS7* Polygenic score including seven SNPs as per Ference et al, *ISH* Ischemic heart disease, *VTE* Venous thromboembolism, *T2D* type 2 diabetes.

^a^adjusted for use of anti-hypertensive medication, as per Ehret et al.

^b^of those who completed the mental health questionnaire.

### PCSK9 locus and CMD

**Fig. 2:**
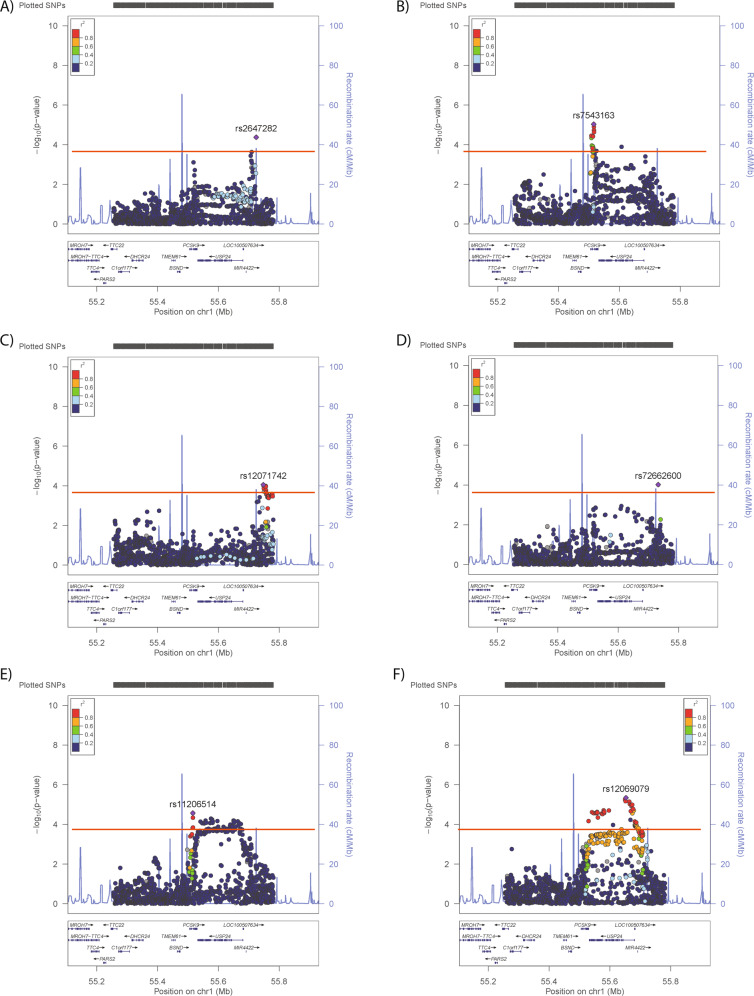
Regional plots of main results in white British ancestry individuals. **A** SBPadj, (**B**) WHRadjBMI, (**C**) VTE, (**D**) stroke, (**E**) mood instability and (**F**) neuroticism score. Locuszoom regional plots of each significant SNP identified for a trait in the UKB. SNPs are aligned on the X-axis by their position on chromosome 9, and by their association with the trait on the Y-axis (*P* values are on a − log10). Significance was set at *p* < 1.16 × 10^−4^ or −log10 *P* > 3.93 (approximated by the red horizontal line). SNPs are colour coded by their estimates of pairwise LD (r2) with lead SNP.

Significant associations are summarised in Table 2 and Fig. 2. One SNP was associated with SBPadj (rs2647282-A, Beta (se) −0.196 (0.048), *p* = 4.20 × 10^−5^, Fig. 2A), eight with WHRadjBMI (lead rs7543163-C, 0.001 (<0.001), *p* = 9.21 × 10^−6^, Fig. 2B) and five with VTE (rs746952505-CA, OR (95% confidence interval) 1.07 (1.04–1.11), *p* = 3.74 × 10^−5^). As the lead SNP for VTE is not available in LocusZoom, the second significant variant was used for plotting and also considered in conditional analyses (rs12071742-A, 1.09 (1.04–1.13), *p* = 8.89 × 10^−5^, Fig. 2C). One SNP was significantly associated with stroke (rs7266260-G, 0.86 (0.80–0.93), *p* = 9.56 × 10^−5^, Fig. 2D). No significant associations were identified with DBPadj, BMI, T2D, ISH or current smoking. Conditional analyses (including the lead variant as a covariate in the analyses) demonstrated that there was only one signal for SBP (Stable 1, SFig 1A, B), WHRadjBMI (Stable 2, SFig 2A, B), VTE (Stable 3, SFig 3A, B, irrespective of which VTE SNP was conditioned on) and stroke (Stable 4, SFig 4A, B).

**Table 2:** Lead SNPs in White British ancestry subset.

Phenotype	CHR	SNP	BP	A1	A2	MAF	*N*	BETA	SE	OR	L95	U95	*P*	N SNPs
SBPadj	1	rs2647282	55,724,437	A	C	0.36	343,109	−0.196	0.048				4.20E-05	1
WHRadjBMI	1	rs7543163	55,515,481	C	T	0.39	400,588	0.001	<0.001				9.21E-06	8
VTE (Lead)	1	rs746952505	55,750,180	CA	C	0.24	264,073			1.07	1.04	1.11	3.74E-05	5
VTE (Proxy)	1	rs12071742	55,747,825	A	G	0.12	277,385			1.09	1.04	1.13	8.89E-05	
Stroke	1	rs72662600	55,732,403	G	A	0.08	339,008			0.86	0.80	0.93	9.56E-05	1
Mood	1	rs11206514	55,516,004	C	A	0.39	377,209			1.02	1.01	1.03	2.68E-05	30
Neuroticism Score	1	rs12069079	55,652,791	G	A	0.20	326,396	0.045	0.010				4.53E-06	48

LD r2 between these 2 SNPs = 0.46.

*A1* Effect or minor allele, *A2* Non-effect allele, *MAF* Effect or minor allele frequency, *N SNPs* N SNPs meeting the threshold for significance, rs746952505 is not available in locuszoom, so for plots rs12071742 is presented as the lead.

### PCSK9 locus and SMI

When considering SMI-related traits (Table 2 and Fig. 2), 30 significant associations were identified for mood instability (rs11206514-C, 1.02 (1.01–1.03), *p* = 2.68 × 10^−5^, Fig. 2E) and 48 for neuroticism (lead rs12069079-G, 0.45 (0.010), *p* = 4.53 × 10^−6^, Fig. 2F). No significant associations were observed for anhedonia, risk-taking behaviour, GAD, MDD, BD or addiction. Conditional analyses demonstrated no additional signals for mood instability (Stable 5, SFig 5A, B) or neuroticism (Stable 6, SFig 6A, B).

### Assessment of independence of signals

Conditional analyses was used to assess whether signals for SMI and CMD traits were independent (ie. represent distinct genetic effects). If there is little or no change in the effect of a trait when adjusting for the lead of a different trait, then signals can be considered independent.

**Fig. 3:**
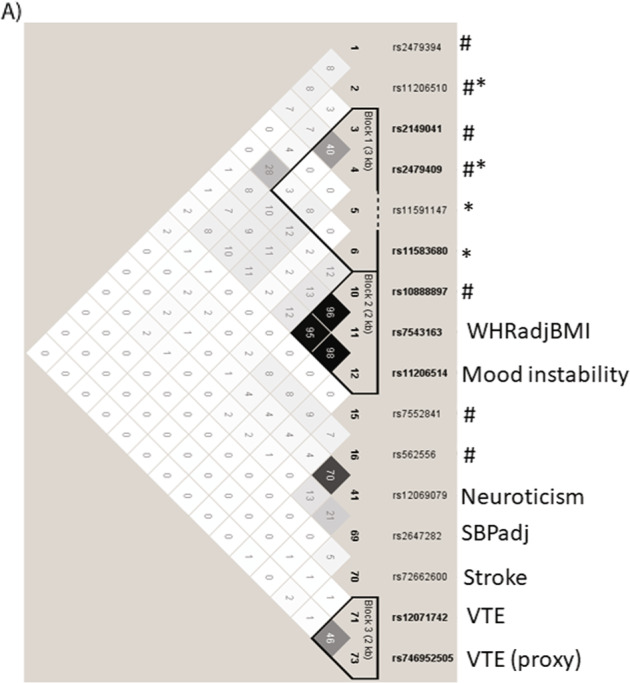
Linkage disequilibrium (LD) between lead SNPs. Haploview LD plot showing the lead SNPs in the *PCSK9* locus associated with cardiometabolic or psychiatric traits, and the SNPs included in PGS4 (*) and PGS7 (#). Each box provides estimated statistics of % frequency of coinheritance (*r*^2^ values). Darker shaded boxes represent stronger LD (*r*^2^ colours).

The lead SNP for SBPadj (rs2647282) demonstrated little change when conditioning on the other lead SNPs (STable 1, SFig 1 C–G), consistent with low LD with other other lead SNPs (max *r*^*2*^ = 0.21, Fig. 3). Effects of WHRadjBMI SNPs were unchanged when conditioning on the lead SNPs for SBP, VTE, stroke or neuroticism score (STable 2, SFig. 2C–E, G) but the association was rendered null by conditioning on the lead SNP for mood instability (SFig. 3F). This is consistent with the very low LD (*r*^*2*^ = 0.4, Fig. 3) with other lead SNPs, but almost complete LD with the mood instability lead SNP (*r*^*2*^ = 0.98, Fig. 3). Of note, the WHRadjBMI-increasing allele had a mood instability-increasing effect (1.02 (1.01–1.03) *p* = 1.50 × 10^−4^). The VTE signal appears to be distinct from other signals, as conditioning on other lead SNPs had little or no impact (STable 3, SFig. 3C–G), consistent with very low LD with other signals (max LD *r*^*2*^ = 0.02, Fig. 3). Similarly, the stroke signal appears independent from the other signals, with conditional analyses having minimal effect (STable 4, SFig 4C–G) and very low LD with any other signal (max *r*^*2*^ = 0.05).

Visual inspection suggests that the signals for mood instability and neuroticism partially overlap (STable 5–6, SFigs 5–6), consistent with the results of the conditional analyses. However, mood instability is a component of the neuroticism phenotype, therefore the conditional analyses likely reflects the phenotypic relationship between these two variables, rather than a shared genetic signal, as the LD between the lead SNPs is very low (LD *r*^*2*^ = 0.04, Fig. 3). of note, the mood instability-increasing allele had a WHRadjBMI-increasing effect (0.001 (<0.001), *p* = 1.25 × 10^−5^).

### Lead SNPs and PCSK9 levels

Of the seven lead/proxy SNPs, five were available in the meta-analysis of PCSK9 levels [24] (Table 3). Nominal (*p* < 0.05) associations were observed for the SNPs associated with SBP (decreasing allele was associated with decreased levels of PCSK9), WHRadjBMI (increasing allele was associated with decreased levels of PCSK9 levels) and mood instability (increasing allele was associated with decreased levels of PCSK9).

**Table 3:** Lookup of lead SNPs in a published meta-analysis of PCSK9 protein levels.

		UKB					Meta-analysis of PCSK9 levels								
Phenotype	SNP	A1	A2	MAF	BETA	OR	EA	NEA	EAF	BETA_a	P_a	I2_a	BETA_b	P_b	I2_b
SBPadj	rs2647282	A	C	0.36	−0.196		A	C	0.37	−0.011	**3.63E-02**	0.57	−0.010	**3.17E-02**	0.55
WHRadjBMI	rs7543163	C	T	0.39	0.001		T	C	0.63	0.019	**6.03E-04**	0.80	0.020	**4.54E-05**	0.89
VTE (Lead)	rs746952505	CA	C	0.24		1.07									
VTE (Proxy)	rs12071742	A	G	0.12		1.09	A	G	0.13	0.002	7.65E-01	0.31	0.006	3.14E-01	0.24
Stroke	rs72662600	G	A	0.08		0.86									
Mood instability	rs11206514	C	A	0.39		1.02	A	C	0.62	0.019	**3.32E-04**	0.77	0.020	**2.35E-05**	0.88
Neuroticism Score	rs12069079	G	A	0.20	0.045		G	A	0.19	−0.006	3.20E-01	0.80	−0.007	1.72E-01	0.80

Bold text indicates nominal significance (*p* < 0.05). Meta-analysis PMID: 34590679

*A1* Effect or minor allele, *A2* Non-effect allele, *MAF* Effect or minor allele frequency, *EA* Effect allele, *NEA* Non-effect allele, *EAF* Effect allele frequency, *a* in individiauls not treated with statins (*n* = 10186), *b* in all individuals, with adjustment for statin use (*n* = 12,721), *I2* Measure of heterogenity across cohorts included in the meta-analysis.

### PCSK9-functional SNPs

To explore whether lipid-lowering functions of PCSK9 might explain the associations observed, we tested two lipid-lowering PGS for impact on our traits of interest (PGS4 [40] and PGS7 [41]). Individual effects of each SNP in the PGS on the traits of interest are presented in STable 7. Only rs10888897 had significant effects on WHRadjBMI (T, 0.001 (<0.001), *p* = 3.75 × 10^−5^), and a non-significant effect on mood instability (T, 1.02 (1.01–1.03) *p* = 0.0003), consistent with high LD between rs10888897 and the lead SNPs for these traits (Fig. 3). The non-significant effect on neuroticism likely reflects phenotypic similarity of these traits, as the LD with the lead neuroticism trait is low. PGS4 and PGS7 showed consistent null effects on SBP, WHRadjBMI, VTE and stroke (*p* > 0.139, STable 8), suggesting a mechanism independent of lipid-lowering. For VTE and stroke, this is consistent effects of *pcsk9* on thrombosis in animal models [42]. PGS7 but not PGS4 had nominally significant (*p* < 0.05) effects on mood instability and both PGS4 and PGS7 had nominally significant (*p* < 0.05) effects on neuroticism, where the direction of effect suggests that lipid-lowering effects of the *PCSK9* locus might contribute to increasing these psychological traits. Whilst rs10888897 is in high LD with the lead SNPs for mood instability (*r*^*2*^ = 0.95, Fig. 3), it is possible that this signal is driving the PRS association with mood instability. The same is not true for the associations of the PGS with neuroticism, as PGS4 contains only SNPs with minimal LD (max *r*^*2*^ = 0.01, Fig. 3) with the lead SNP for neuroticism (and max *r*^*2*^ = 0.12 with the lead SNP for mood instability).

### Secondary analyses

Analyses of additional ancestry groups were restricted to phenotypes significant in discovery analyses with sufficient data. No SNPs met the thresholds for significance in any analyses (neuroticism, mood instability, SBPadj or WHRadjBMI in all groups or VTE in white European ancestry). Lead SNPs for secondary analyses (reaching *p* < 1 × 10^−3^) are presented in STable 9 and a comparison of effect directions of the lead SNPs from the discovery analysis, across ancestry groups is provided in STable 10. The lack of significant findings in non-white British ancestry individuals is likely due to underpowered analyses. However, LD between the lead SNPs in the non-white British ancestry individuals confirmed the conclusion that the signals for WHRadjBMI and mood instability overlap (also with one lipid-lowering variant), but the other signals are independent (SFig 7).

### Follow-up analyses

PCSK9 protein expression was detected in both the liver and brain. mRNA expression of *PCSK9* in systems essential to CMD and SMI pathology is striking (Fig. 1A). Expression of two other genes in this locus is worth noting: *DHCR24* (Fig. 1B) is predominantly expressed in the adrenal gland and spinal cord and *USP24* (Fig. 1C) is ubiquitously expressed. The lead SBPadj SNP demonstrated genotype-specific gene expression patterns in heart, nerve and muscle tissues, but for *USP24*, not *PCSK9* (Table 4). No other lead SNP demonstrated effects on *PCSK9* mRNA expression. Proxies of the WHRadjBMI lead SNP showed genotype-specific gene-expression for *DHCR24* in brain tissue, *USP24* in heart tissue and *PCSK9* in brain and adipose tissues, where the WHRadjBMI-increasing allele, also associated with decreased PCSK9 protein levels, was associated with decreased expression in the brain and increased expression in adipose tissue. The disconnect between (some) tissue mRNA expression levels and circulating levels of PCSK9 protein, has previously been reported [43]. The opposite direction of effects of one SNP in the brain vs adipose tissue is curious and may suggest that the balance of *PCSK9* and therefore lipids, between the brain and adipose tissues contributes to mood instability and central fat accumulation (as indicated by WHRadjBMI).

**Table 4:** Genotype-specific gene expression of trait-associated SNPs or proxies (LD R2 > = 0.50).

Trait	Lead SNP	Proxy SNP	Position	R2	D'	Gene	Tissue	EAF	Effect	*P* value
SBP	rs2647282		55,724,437			*MROH7*	Esophagus-Mucosa	*A* = 0.324	−0.119	1.11E-04
						*USP24*	Heart-Atrial Appendage	*A* = 0.324	−0.139	3.08E-06
						*USP24*	Muscle-Skeletal	*A* = 0.324	−0.080	9.75E-06
						*USP24*	Nerve-Tibial	*A* = 0.324	−0.093	1.43E-04
						*USP24*	Skin-Sun Exposed (Lower leg)	*A* = 0.324	−0.115	5.28E-07
						*USP24*	Skin-Not Sun Exposed (Suprapubic)	*A* = 0.324	−0.185	2.44E-11
WHRadjBMI	rs7543163	rs11206513	55,507,649	0.915	1.000	*DHCR24*	Brain-Cortex	*T* = 0.544	0.129	5.06E-05
		rs11206514	55,516,004	1.000	1.000	*DHCR24*	Brain-Cortex	*A* = 0.566	0.128	4.83E-05
		rs11436234	55,511,623	0.893	0.977	*DHCR24*	Brain-Cortex	TC = 0.549	0.136	2.47E-05
		rs7530425	55,511,471	0.915	1.000	*DHCR24*	Brain-Cortex	*T* = 0.544	0.136	2.47E-05
		rs10888896	55,509,213	0.579	1.000	*PCSK9*	Adipose-Visceral (Omentum)	*C* = 0.692	−0.277	2.66E-05
		rs10888898	55,516,508	0.978	1.000	*PCSK9*	Skin-Not Sun Exposed (Suprapubic)	*G* = 0.56	−0.138	9.28E-05
		rs2479410	55,505,861	0.619	0.814	*PCSK9*	Brain-Cerebellum	*A* = 0.418	−0.369	9.61E-05
						*PCSK9*	Skin-Not Sun Exposed (Suprapubic)	*A* = 0.418	0.162	1.73E-05
		rs644000	55,511,995	0.694	0.902	*PCSK9*	Skin-Not Sun Exposed (Suprapubic)	*G* = 0.396	0.152	4.58E-05
		rs6681159	55,507,882	0.579	1.000	*PCSK9*	Adipose-Visceral (Omentum)	*T* = 0.692	−0.255	5.79E-05
		rs7543163	55,515,481	1.000	1.000	*USP24*	Heart-Atrial Appendage	*T* = 0.566	0.116	8.22E-05

Proxies are defined as SNPS in at least moderate LD (LD R2 > = 0.50). Variant positions are given aligned to GRCH38.

VEP highlighted rs487230 (significant for mood instability and neuroticism) as a tolerated/benign missense variant. This SNP had effects on *USP24* in many tissues, where the mood instability/neuroticism-increasing allele was associated with reduced expression. No other trait-associated SNPs were predicted to have functional effects.

Our results do not contradict the observation that PCSK9-associated genetic variants reported so far have loss-of function effects (reduced expression or function), with the only positive effect being attributed to compensation for reduced protein function [24].

### Comparisons with previous studies

The lead SNPs reported here have not previously been robustly associated with any trait. STable 11 summarises previous associations in the *PCSK9* locus, of which many are for CMD traits. It is noteworthy that there were no previous associations of *PCSK9* SNPs with VTE or psychiatric traits in GWAS. (STable 11).

Compared to the phenome-wide scan of rs11591147 [23], the present study included unrelated individuals of white British ancestry, rather than all participants. Results are broadly similar with blood pressure and obesity (SBPadj and WHRadjBMI) associations. The discrepancy in T2D likely reflects differences in phenotyping (self-report vs self-report plus medication).

## Discussion

This systematic analysis of the *PCSK9* locus for effects on CMD and SMI-related traits in UK Biobank identified signals associated with four cardiometabolic traits (VTE, stroke, SBPadj and WHRadjBMI) and two psychological traits (mood instability and neuroticism). We demonstrated that the WHRadjBMI and mood instability signals overlap, that the effects of *PCSK9* on the SMI (but not CMD) likely act through lipid-lowering mechanisms. Finally, we present evidence that these SNP regulate mRNA expression levels of *PCSK9, DHCR24* and *USP24*.

To our knowledge this is the first study to report an effect of the *PCSK9* locus, specifically through lipid-lowering effects, on psychological phenotypes (mood instability and neuroticism) of relevance to psychiatric traits. The associations with SBPadj and WHRadjBMI were unsurprising, however, it is unusual that SBPadj, but not DBPadj, was significantly associated with this locus. The association of the *PCSK9* locus with VTE was unexpected, given that lipid accumulation and inflammation are generally considered less relevant to VTE than arterial disease. However, a non-lipid-lowering mechanisms is plausible, as *pcsk9*-knockout in experimental animals reduced platelet activation [42], likely via clearance of clotting factor VIII by the LDLR family. This is consistent with our finding that the same allele of the VTE proxy (rs12071742-A) both increases VTE risk and PCSK9 levels (Table 3). A detailed summary of PCSK9 effects (and their inhibition) on platelet activation has been reported [13], with recent evidence for platelet-derived PCSK9 adding complexity [44].

The almost complete LD reported here between the lead variants for mood instability and WHRadjBMI is intriguing: does mood instability subsequently influence central fat accumulation (through diet and exercise preferences for example), or does this genetic signal have multiple effects on different genes and in different tissues (pleiotropy), or is there one mechanism common to these two very different traits? The gene expression evidence of the minor allele (associated with increased WHRadjBMI and decreased PCSK9 protein levels) both increasing *PCSK9* levels in adipose tissue and decreasing *PCSK9* levels in brain tissue suggests that looking at one system in isolation might not be enough.

The non-white British ancestry results were largely uninformative (due to limited power), however the consistent LD patterns across ancestry groups supports the overlap of signals for mood instability and central fat accumulation (SFig. 7).

In addition to *PCSK9*, expression data highlights the roles of *DHCR24* and *USP24* in SMI and CMD: DCHR24 has been shown to have a neuroprotective role during inflammation [45] with loss-of *DCHR24* expression being observed in Alzheimer’s disease [45, 46]. WHRadjBMI-increasing alleles being associated with reduced *DHCR24* levels in the brain could suggest the same mechanisms underly central adiposity and lack of neuroprotection. *USP24* is implicated in Parkinson’s disease and increased autophagy [47], but expression data presented here indicate a role for *USP24* in heart, nerve, and muscle tissue, but not adipose or brain tissues.

**Fig. 4:**
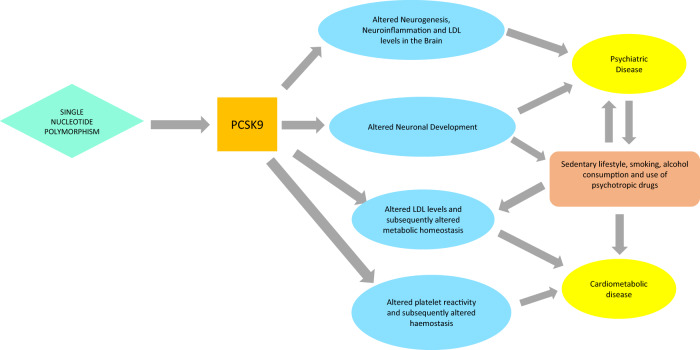
Potential *PCSK9* mechanisms proposed pathways of how genetic variation in the *PCSK9* locus might influence cardiovascular and metabolic traits.

Fig. 4 shows some of the potential mechanisms and pathways by which PCSK9 could contribute to SMI and CMD. Further studies are required to elucidate which of these (and/or other) pathways are most important to SMI, CMD and their comibidity, and whether there is potential for repurposing PCSK9-inhibitors (currently used for CMD) for improving SMI symptoms and CMD prevention.

### Strengths and limitations

SMI and CMD and their comorbidity are increasingly recognised as an important individual, societal and economic burden, therefore this study is very relevant. The large sample size and consistent phenotyping in the UKB is a strength, as is the conservative Bonferroni correction, both of which contribute to the reliability of these findings. The PGS provided additional depth to mechanistic insights of this study. The biases of UKB are well established {Swanson, 2012 #268}, and self-report to assess psychiatric traits could provide additional biases (for example, mood instability and risk taking: Individuals with SMI may not regard their behaviour as ‘risky’ or recognize their mood to fluctuate). Therefore, UKB likely underestimates effects of SNPs on disease. Further exploration of these findings in additional ancestry groups would be of great value.

## Conclusion

In a systematic analysis of the *PCSK9* locus, we identified genetic associations with SBPsdj, WHRadjBMI, VTE, stroke, mood instability and neuroticism score, with the same signal (indicated by LD *r*^*2*^ = 0.98) associated with WHRadjBMI and mood instability. This is the first study to implicate the *PCSK9* locus in the shared pathology of CMD and SMI. The lipid-lowering PGS demonstrating a nominal association with neuroticism score and mood instability highlights important considerations for lipid-lowering drugs. Subsequent analyses should consider causal assessments and temporal patterns of *PCSK9*, *DCHR24* and *USP24* expression, to elucidate the mechanisms linking CMD and SMI. Further exploration of this locus in a more diverse cohort would improve understanding of the locus and health equality in the future.

## Supplementary information

Supplemental Figures

Supplemental Table 1

Supplemental Table 2

Supplemental Table 3

Supplemental Table 4

Supplemental Table 5

Supplemental Table 6

Supplemental Table 7

Supplemental Table 8

Supplemental Table 9

Supplemental Table 10

Supplemental Table 11

## Data Availability

All UK Biobank data (raw, coding and results) are available to approved researchers via application to the UK Biobank study (https://www.ukbiobank.ac.uk/). Coding and summary level results are available upon request (Dr Rona J Strawbridge, rona.strawbridge@glasgow.ac.uk).
