# Supplementary material for: Genetic analysis of the PCSK9 locus in psychological, psychiatric, metabolic and cardiovascular traits in UK Biobank
Source: Eur J Hum Genet. 2022 May 2;30(12):1380–90. doi: 10.1038/s41431-022-01107-9 (PMC9712543; doi:10.1038/s41431-022-01107-9)
Supplement: Supplementary file 1 — Supplemental Figures [file 41431_2022_1107_MOESM1_ESM.pdf]

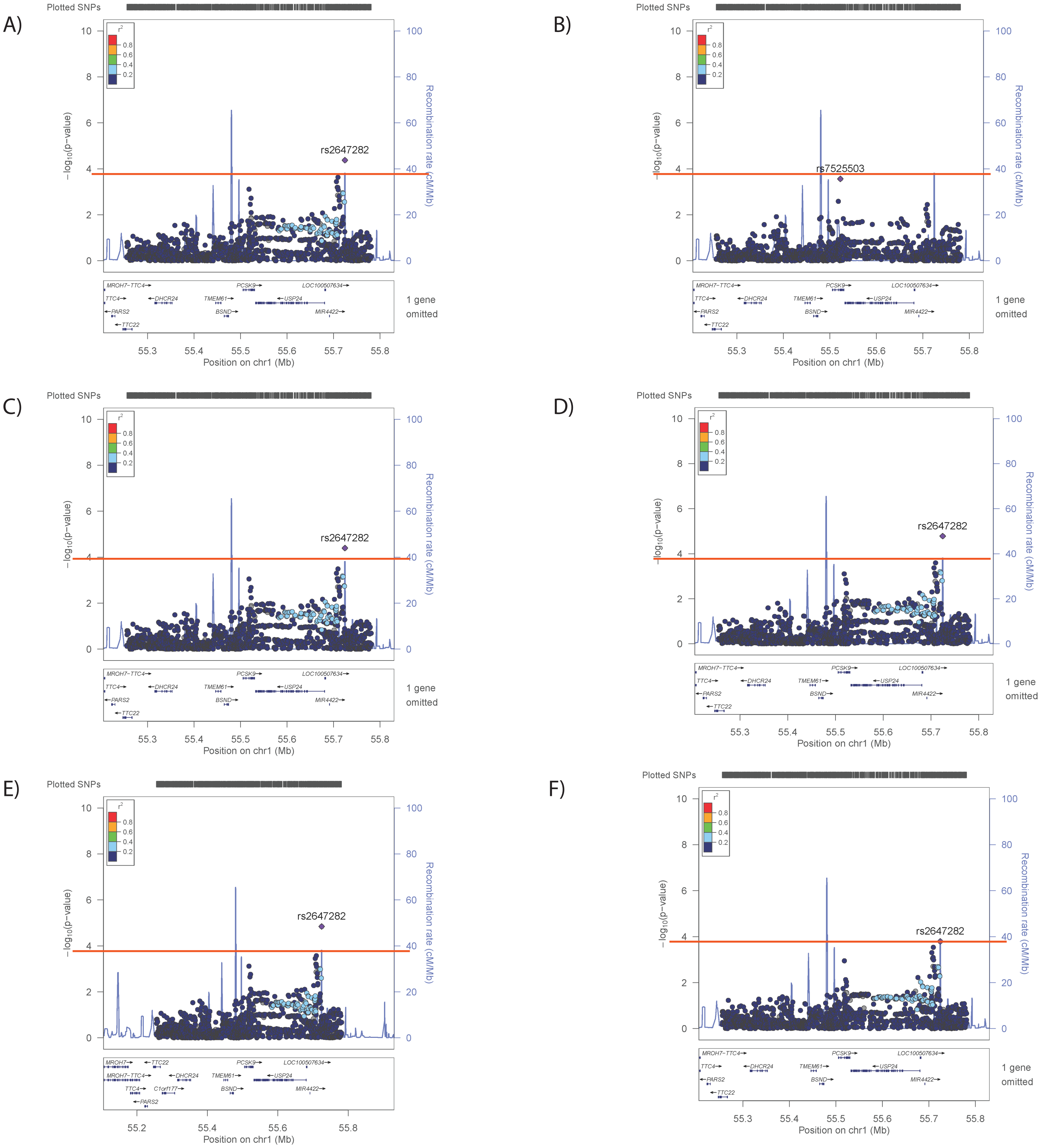

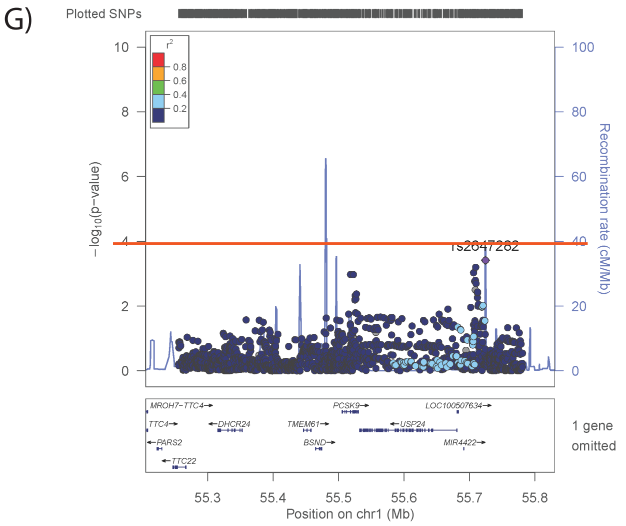

Supplemental Figure 1: Regional plots for the association of the PCSK9 locus with A) SBPadj, conditioned on the lead SNP for B) SBPadj, C) WHRadjBMI, D) VTE, E) stroke, F) mood instability and G) neuroticism

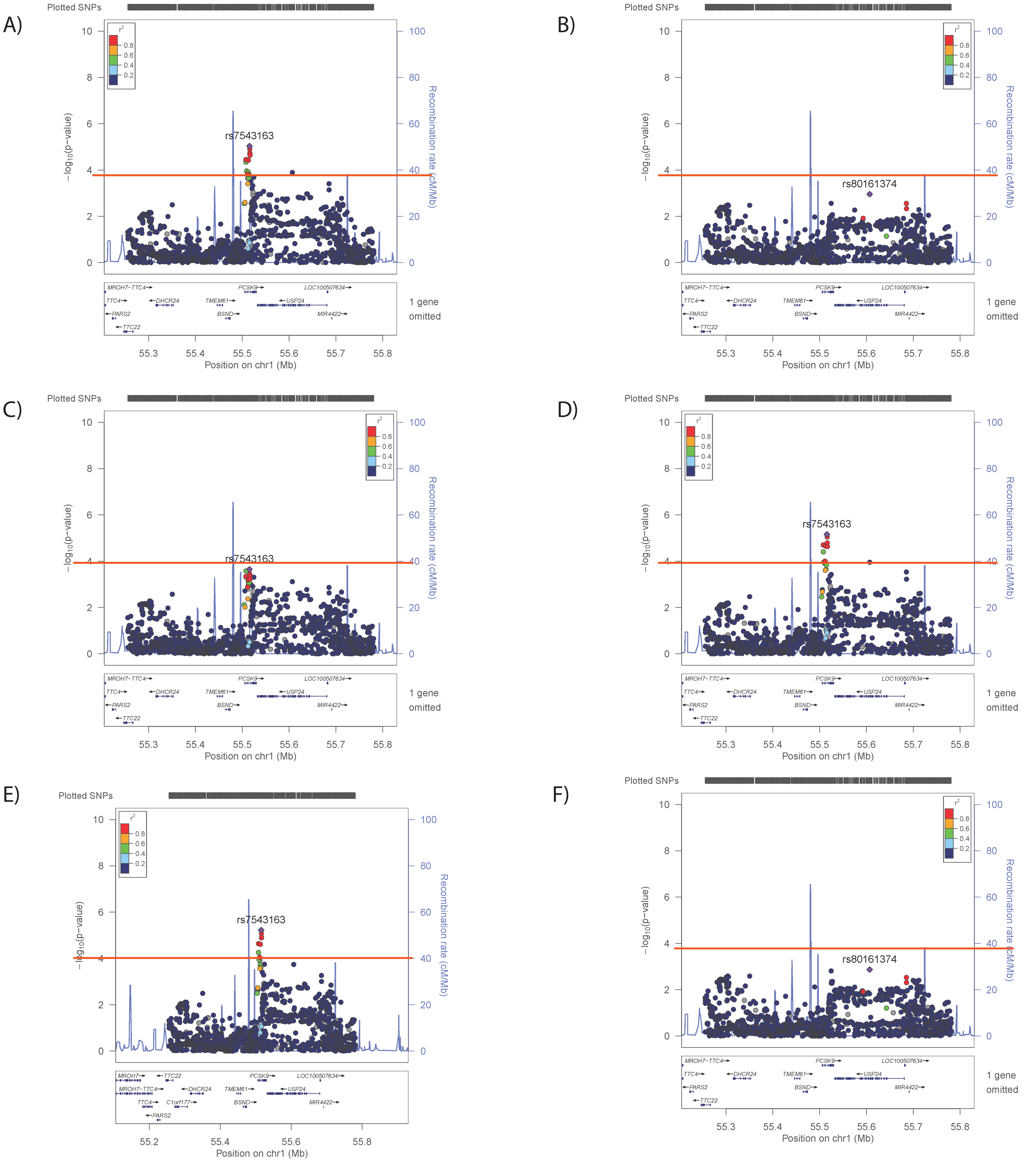

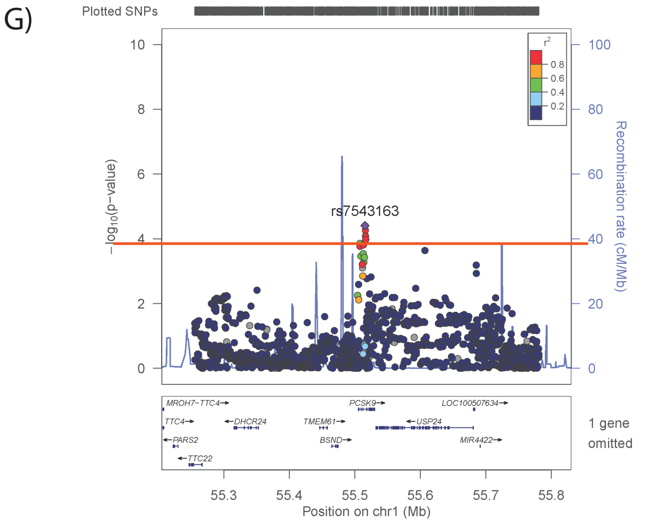

Supplemental Figure 2: Regional plots for the association of the PCSK9 locus with A) WHRadjBMI, conditioned on the lead SNP for B) WHRadjBM, C) SBPadj, D) VTE, E) stroke, F) mood instability and G) neuroticism

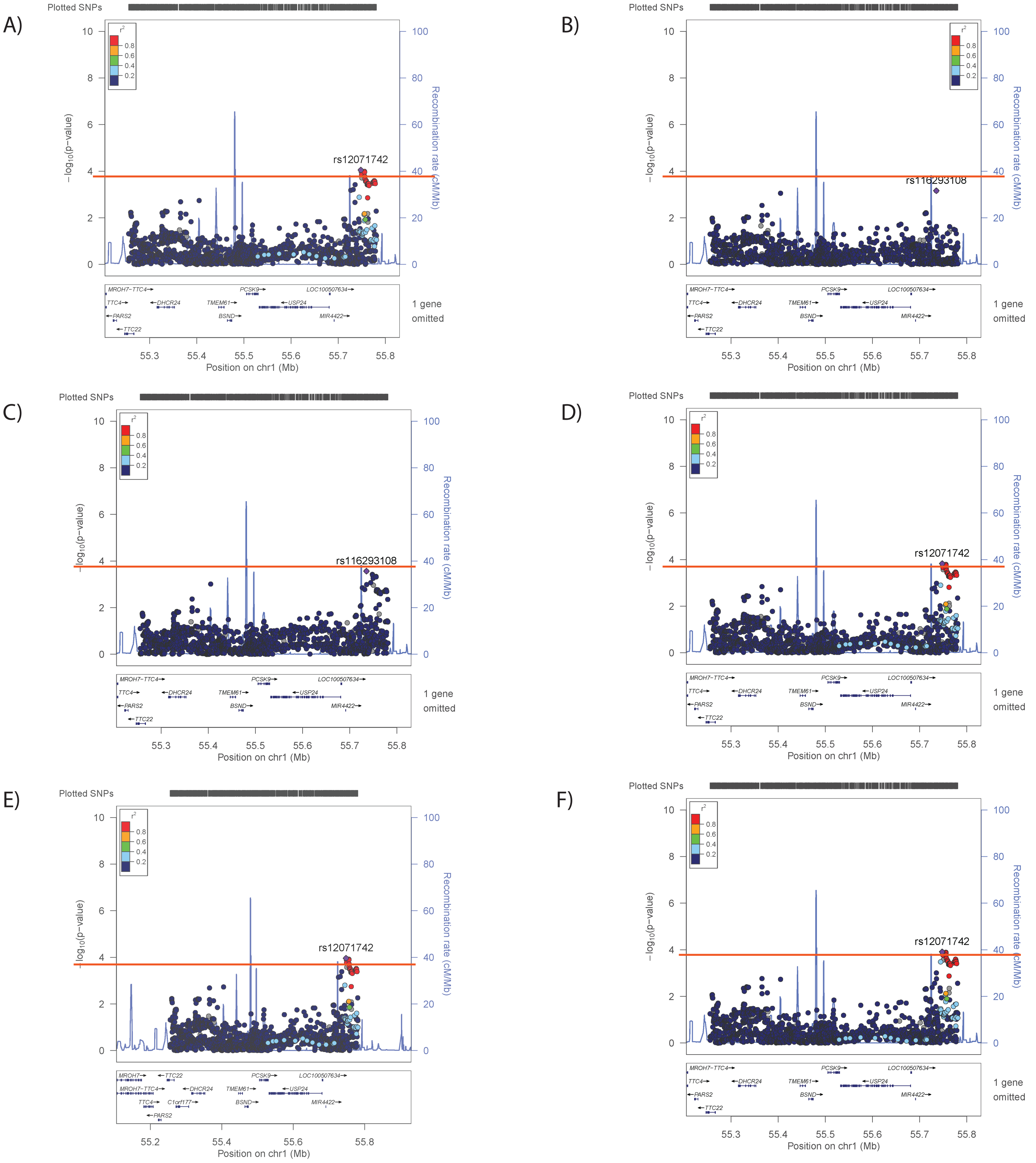

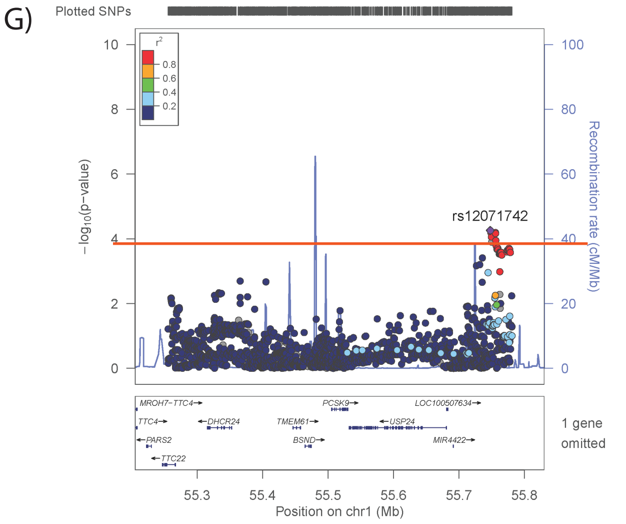

Supplemental Figure 3: Regional plots for the association of the PCSK9 locus with A) VTE, conditioned on the lead SNP for B) VTE C) SBPadj, D) WHRadjBMI, E) stroke, F) mood instability and G) neuroticism

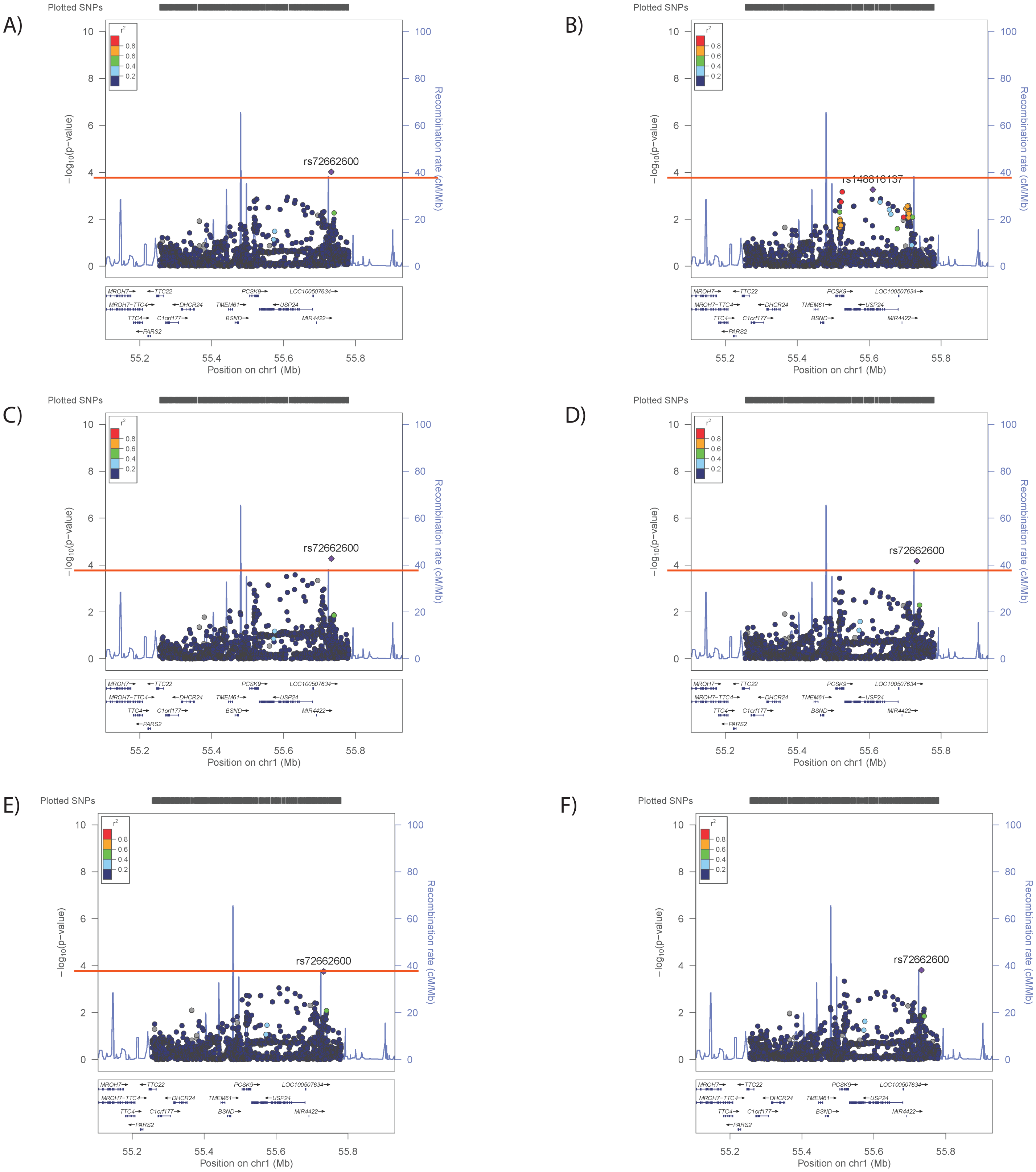

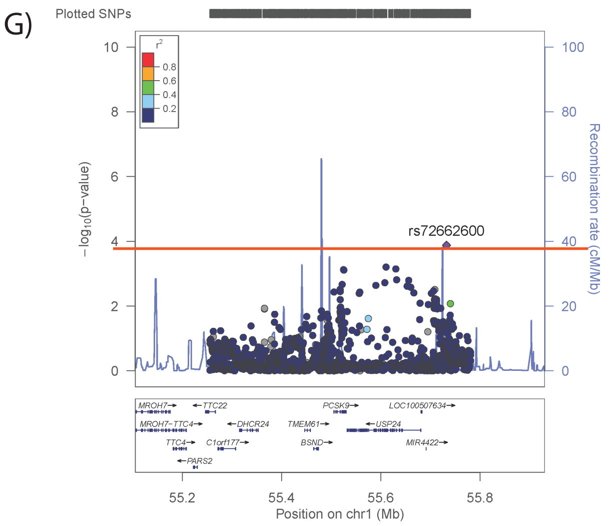

Supplemental Figure 4: Regional plots for the association of the PCSK9 locus with A) stroke, conditioned on the lead SNP for B) stroke, C) SBPadj, D) WHRadjBMI, E) VTE and F) mood instability, G) neuroticism

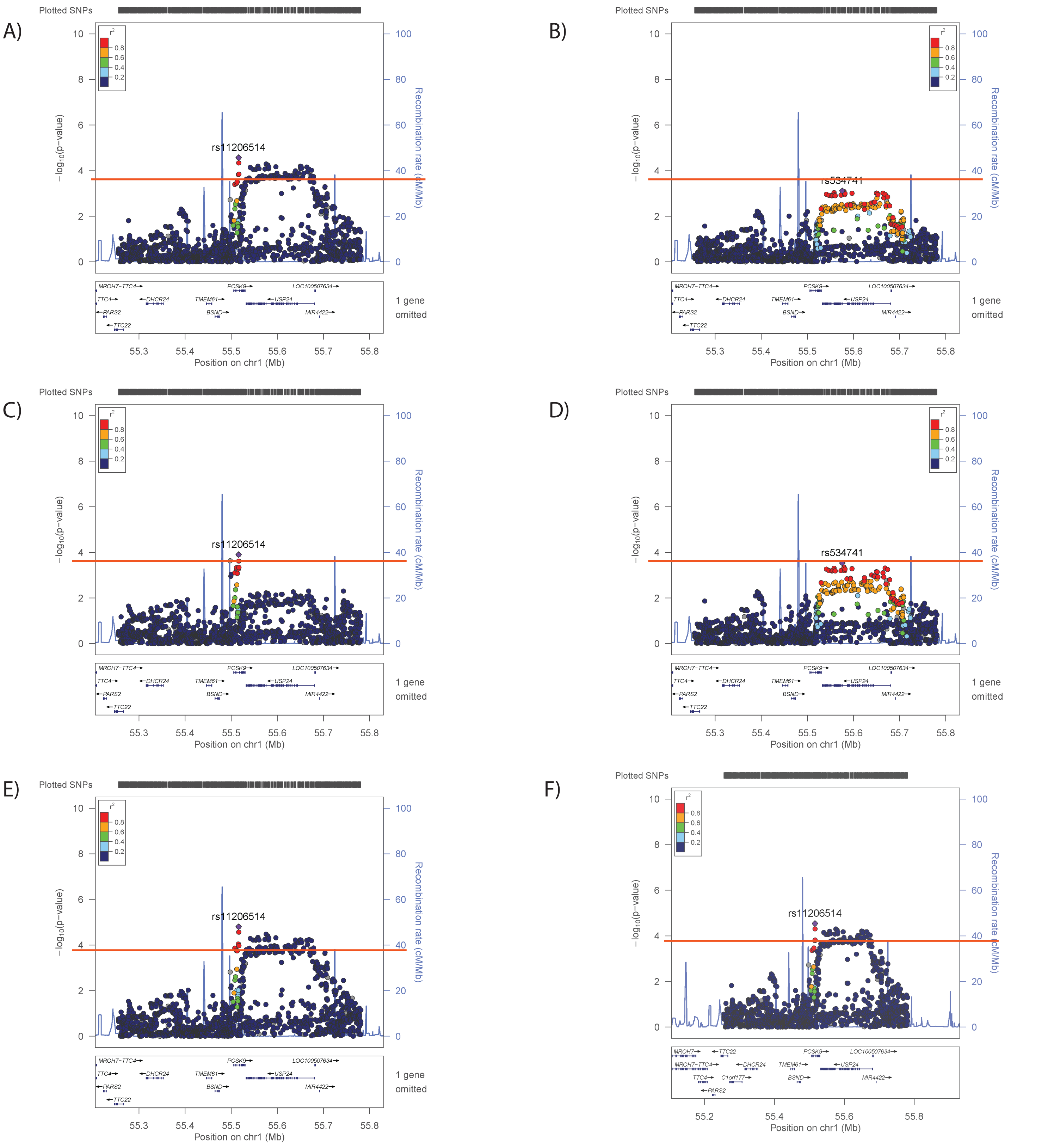

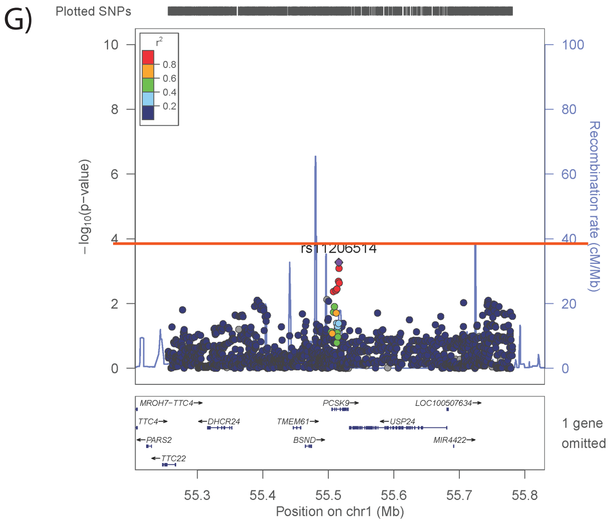

Supplemental Figure 5: Regional plots for the association of the PCSK9 locus with A) mood instability, conditioned on the lead SNP for B) mood instability, C) SBPadj, D) WHRadjBMI, E) VTE, F) stroke, G) neuroticism

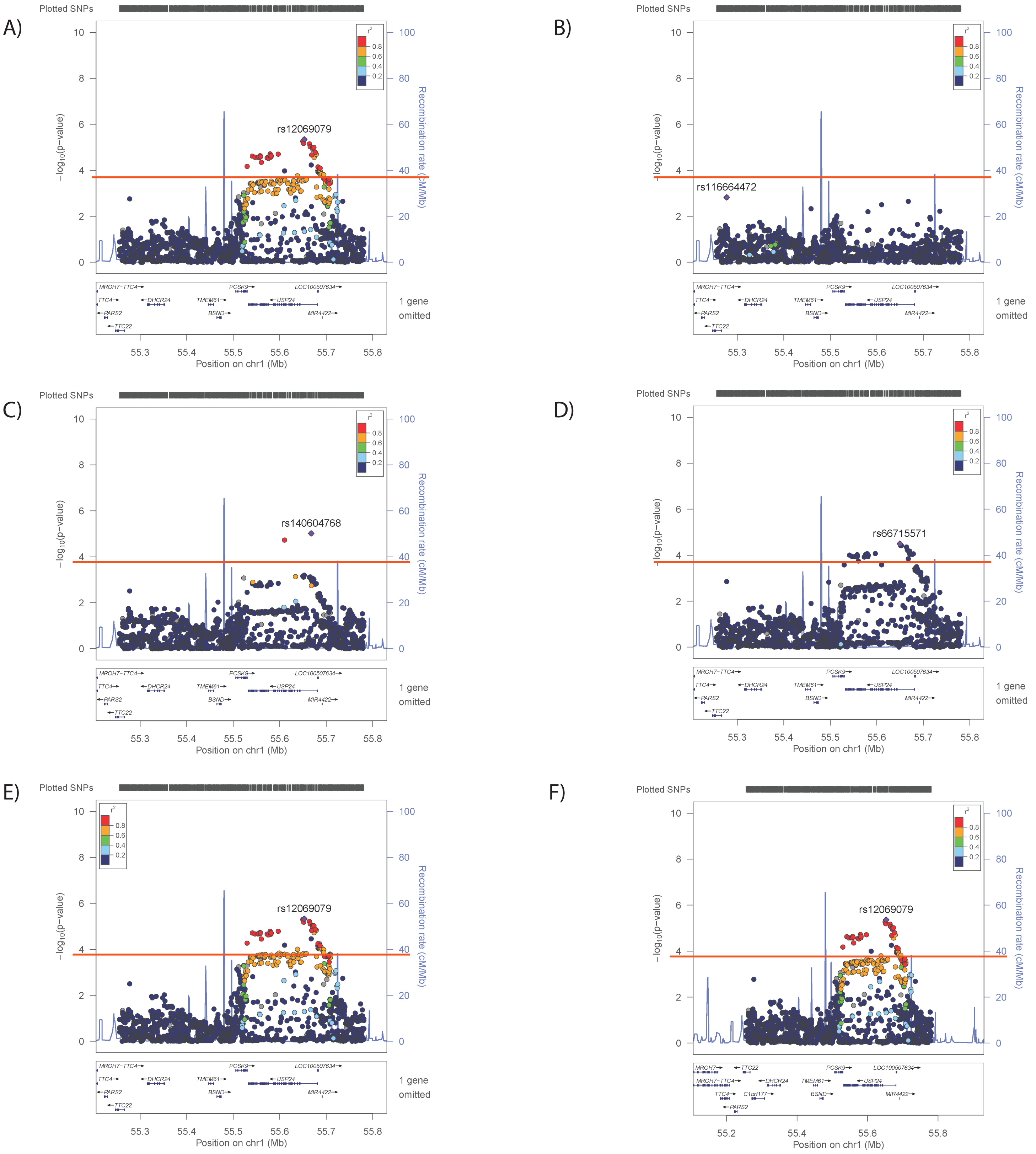

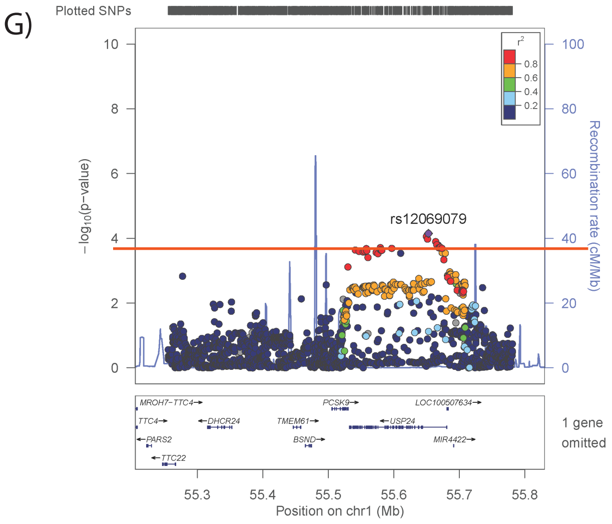

Supplemental Figure 6: Regional plots for the association of the PCSK9 locus with A) neuroticism, conditioned on the lead SNP for B) neuroticism, C) SBPadj D) WHRadjBMI, E) VTE, F) stroke, G) mood instability

A)

B)

C)

D)

E)

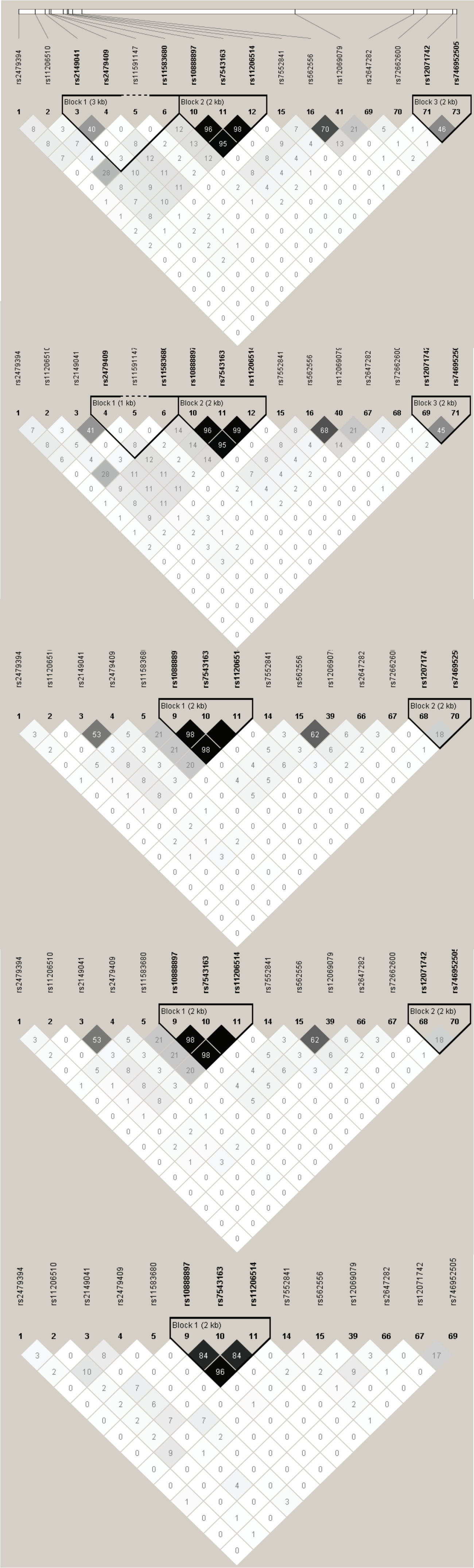

Supplementary Figure 7: LD plots of significant SNP or their proxies in individuals of A) white British ancestry, b) white European ancestry, C) south Asian ancestry, D) Mixed ancestry and E) African-Caribbean ancestry.
